# Protagon

**Published:** 1868-07

**Authors:** 


### ARTICLE IV.

### *Protagon.*

All our readers who have studied Physiological Chemistry are aware that the chemistry of the Brain has been for a long time in an unsettled and unsatisfactory condition. Certain excrementitious products were constantly found in it, but it was impossible to trace these to their source. Large quantities of phosphates and free phosphoric acid were known to exist in its ashes, and when it was overworked, quantities of phosphates were found to make their appearance in the urine. From this it was naturally inferred that the natural, active substance of the brain was a phosphorized material which, undergoing change, had phosphoric acid as the necessary product of its oxidation. This was sought in various phosphorized fats about which chemists have written so much. Unfortunately, however, there was no agreement among observers. Every man who made a new study of the chemistry of the brain, did no more than overthow the labors of his predecessors.

Thus up to 1840, Couerbe's opinion was universally received. He thought that the brain contained cholesterine and distinct fat-like bodies. In that year, Payen completely overturned his theory, showing that these fat-like bodies were only mixtures of known fats with albuminates and two acids which he first described as *cerebric* and *oleophosphoric*. Fr. Müller subsequently obtained from the brain a white nitrogenous substance, free from sulphur and phosphorus, which he called cerebrine.

We are now at the commencement of a new revolution. Three years ago, Liebreich announced that scarcely any of these fat-like matters has any real existence in the living brain, but that they are all products of decomposition of a single essential substance which he calls *Protagon.*

After thoroughly washing the brain through the vessels, slicing and frequently washing with water and ether at 32° F. the latter menstrum being needed to remove the cholesterine, the protagon may be extracted from the nearly pure brain-pulp, by 85 per cent alcohol at 112° F. On cooling this solution to 32°F. there falls an abundant flocculent precipitate which is washed with ether at 32° F, as long as cholesterine is extracted. The protagon may then be dissolved in dilute warm alcohol, from which it separates on cooling, pure, in snow-white acicular prisms of microscopic minuteness, arranged in radiating and star-like groups.

Liebreich's analysis of this compound is

| | |
|---|---|
| Carbon | 66.7 |
| Hydrogen | 11.7 |
| Nitrogen | 2.8 |
| Phosphorus | 1.2 |
| Oxygen | 17.6 |
| | 100.0 |

and his formula $C_{232}\ H_{240}\ P\ O_{44}$ in which C=6 and O=8, the old numbers. It swells very much in water, forming a translucent starch-like paste, and requires a very large quantity of water to dissolve it. It dissolves in strong acetic acid and in alcohol a little below 212° F.; heat reduces it to a soft plastic mass. Boiled for 24 hours with saturated baryta water, it breaks up into *glycero-phosphoric acid*, a strongly alkaline base which Liebreich calls *neurine*, and *fatty acids*. The formulæ for these substances are, glycero-phosphoric acid, $C_6\ H_9\ PO_{12}$, neurine, $C_{10}\ H_{13}\ N$. He expresses the reaction with baryta water by the following formula.

Protagon, $C_{232}\ H_{240}\ N_4\ PO_{44}=4\ (C_{10}\ H^{13}\ N)$, Neurine, + $C_6\ H_9\ PO_{12}$, Glycero-phosphoric acid, + $C_{186}\ H_{179}\ O_{32}$, remainder, consisting of fatty acids, stearic acids &c.

These decompositions lead to the belief that Fremy's cerebric acid is a mixture of protagon and fatty acids, and that Muller's neurine is nitrogenous neurine combined with a non-nitrogenous fatty acid. The fact that it yields glycerine seems to account for the presence of various fatty and other acids in the brain. Certain recently observed optical phenomena of the nerves appear to point to the presence of protagon in the medullary matter.

Myeline, which is found in many structures, and is closely allied to nervous medullary matter, has been readily obtained from protagon by Liebreich who thinks that it is a mixture of unchanged protagon with some of the products of its decomposition. According to these views, the brain may be regarded as essentially composed of protagon, albuminates and cholesterine, with unimportant traces of other organic matters.

Nor has this substance been found in the brain only. It has been detected in the corpuscles of the blood both red and white, in pus-corpuscles, and in pulmonary tissue.

Whether these views which so simplify our notions of the constitution of nervous matter are to share the fate of so many of their predecessors, or whether they are the precursors of a new and true physiological chemistry, it would be at present premature to decide.

---

